# Quantification of heterotypic granule fusion in human neutrophils by imaging flow cytometry

**DOI:** 10.1016/j.dib.2015.12.003

**Published:** 2015-12-17

**Authors:** Halla Björnsdottir, Amanda Welin, Claes Dahlgren, Anna Karlsson, Johan Bylund

**Affiliations:** a Department Oral Microbiology and Immunology, Institute of Odontology, Sahlgrenska Academy, University of Gothenburg, Sweden; b Department Rheumatology and Inflammation Research, Institute of Medicine, Sahlgrenska Academy, University of Gothenburg, Sweden

## Abstract

Human neutrophils are filled with intracellular storage organelles, called granules and secretory vesicles, which differ in their content of soluble matrix proteins and membrane-bound molecules. To date, at least four distinct granule/vesicle subsets have been identified. These organelles may secrete their content extracellularly following mobilization to and fusion with the plasma membrane, but some of them may also fuse with internal membrane-enclosed organelles, typically a plasma membrane-derived phagosome. There are also instances where different granules appear to fuse with one another, a process that would enable mixing of their matrix and membrane components. Such granule fusion enables e.g., myeloperoxidase-processing of intragranular oxygen radicals, a key event in the formation of neutrophil extracellular traps (Björnsdottir et al., 2015) [1]. Described herein are data that show the quantification of such heterotypic granule–granule fusion by the use of imaging flow cytometry, a technique that combines flow cytometry with microscopy. The analysis described is based on immunofluorescent staining of established granule markers (lactoferrin and/or NGAL for one granule subset; the specific granules, and CD63 for another granule subset, the azurophil granules) and calculation of a colocalization score for resting and PMA-stimulated neutrophils.

**Specifications Table**

**Table:** 

Subject area	*Biology*
More specific subject area	*Phagocyte biology*
Type of data	*Imaging flow cytometry data, image analyses*
How data was acquired	*Imaging flow cytometry; ImageStreamX MkII*
Data format	*Analyzed data, raw data (.cif and .daf files from one representative experiment)*
Experimental factors	*Isolated human neutrophils were kept on ice or treated with PMA for 3 min at 37 °C. Samples were fixed, permeabilized and immunostained for granule markers before analysis.*
Experimental features	*The degree of colocalization between the different granule markers was quantified by the IDEAS software.*
Data source location	*Gothenburg, Sweden*
Data accessibility	*Supplementary data*

**Value of the data**

• Imaging flow cytometry provides means to objectively quantify the intensity and distribution of fluorescent probes.
• Combined with immunostaining for established neutrophil granule markers, imaging flow cytometry enables unbiased quantification of marker colocalization, indicative of heterotypic granule–granule fusion.
• The data provide insights into the dynamic trafficking of granules in human neutrophils and present a valuable method to study this phenomenon.

## 1 Data

Human neutrophils are equipped with storage organelles, so-called granules, for a wide variety of molecules needed during the limited life-span of these cells [2]. To date, at least 4 distinct organelle types are recognized based on content, time of formation during neutrophil maturation in the bone marrow, as well as propensity to be mobilized. First to be formed, and least likely to be mobilized to the plasma membrane, are the azurophil granules that contain a wide variety of antimicrobial effector molecules. Next to be formed are the specific and gelatinase granules, storing various receptors and matrix metalloproteinases of importance for extravasation of the cells from blood to tissue. Finally, the secretory vesicles are formed in mature neutrophils, [3] shortly before their exit from the bone marrow into the blood. Whereas secretory vesicles and gelatinase granules can be mobilized to the plasma membrane with relative ease (resulting in secretion of matrix components), the specific granules and azurophil granules are more likely to fuse with internal membranes, most typically the membrane of a phagosome [4]. In order to study these subcellular organelles, there are plenty of distinct granule markers available, which are more or less specifically expressed in a single type of granule [5].

When neutrophils are stimulated they typically assemble and activate the superoxide-generating NADPH-oxidase resulting in the production of reactive oxygen species (ROS). The NADPH-oxidase is a multi-component enzyme system consisting of membrane-bound as well as cytosolic components [6]. The membrane-bound components are present in the plasma membrane, but mainly expressed in the membranes of gelatinase granules and specific granules [7]. In contrast, the enzyme myeloperoxidase (MPO) which converts primary ROS (superoxide and hydrogen peroxide) to even more reactive secondary metabolites, is specifically stored within azurophil granules [8]. During phagocytosis of a particulate prey both specific granules and azurophil granules fuse with the phagosome so that the primary ROS stemming from the NADPH-oxidase may be properly processed by MPO within this mature so-called phagolysosome [4]. However, also in the absence of phagocytosis, stimulated neutrophils may produce MPO-processed ROS within intracellular compartments [9], [10], [11], [12], [13], [14], [15] and we have previously hypothesized that this is the result of heterotypic granule–granule fusion between specific granules (containing an active NADPH-oxidase) and azurophil granules (containing MPO), giving the primary ROS and MPO a chance to interact [16], [17], [18]. One soluble stimulus that potently evokes the production of MPO-processed ROS in granules is phorbol myristate acetate (PMA) [19], a synthetic activator of protein kinase C, but there are also numerous examples of naturally occurring activators [9], [11], [13], [14]. The functional role of the intra-granular MPO-processed ROS is largely unexplored, but we have very recently demonstrated that they are in fact imperative for PMA-triggered formation of neutrophil extracellular traps [1].

In the data presented (Supplemental data), we utilize immunofluorescent staining of established granule markers; CD63 for azurophil granules and lactoferrin or NGAL for specific granules [5]. Stimulated, permeabilized and thereafter stained cells were subjected to imaging flow cytometry, and heterotypic granule fusion, i.e., the degree of colocalization of these markers, was calculated using the built-in colocalization analysis of the IDEAS software. Multiple gating steps were needed prior to applying the colocalization analysis and below follows a detailed description of how to prepare and analyze the samples.

## 2 Experimental design, materials and methods

### 2.1 Experimental setup

Human granulocytes were isolated from buffy coats as described by Bøyum [20], and diluted to 1×10^7^ cells/ml in Krebs-Ringer phosphate buffer (KRG) containing glucose (10 mM), Mg^2+^ (1.5 mM) and Ca^2+^ (1 mM). Cells were either kept on ice or stimulated with PMA (50 nM) for 3 min at 37 °C. Indirect immunofluorescent staining of cells was performed essentially as described in [21]. In short, cells were fixed and permeabilized with a fixation/permeabilization kit (BD) and incubated with combinations of the following primary antibodies; lactoferrin (DAKO, 89 μg/ml), CD63 (Sanquin, 2 μg/ml), and/or NGAL (Abcam, 5 μg/ml). Samples were then incubated with secondary antibodies (F(ab׳)_2_ fragments; 4 μg/ml) conjugated with Alexa Fluor 488 or 647 in combinations as is indicated in the figures. Samples were diluted in 20 μl PBS and supplemented with DAPI (3 μM) for staining of DNA. The cells were analyzed in an imaging flow cytometer (ImageStreamX MkII) using the 60× objective and the IDEAS software (v. 6.1, Amnis) was used for analysis. At least 3000 events per sample were collected. The raw data from one representative experiment can be found as Supplemental data.

### 2.2 Gating strategy

**Fig. 1:**
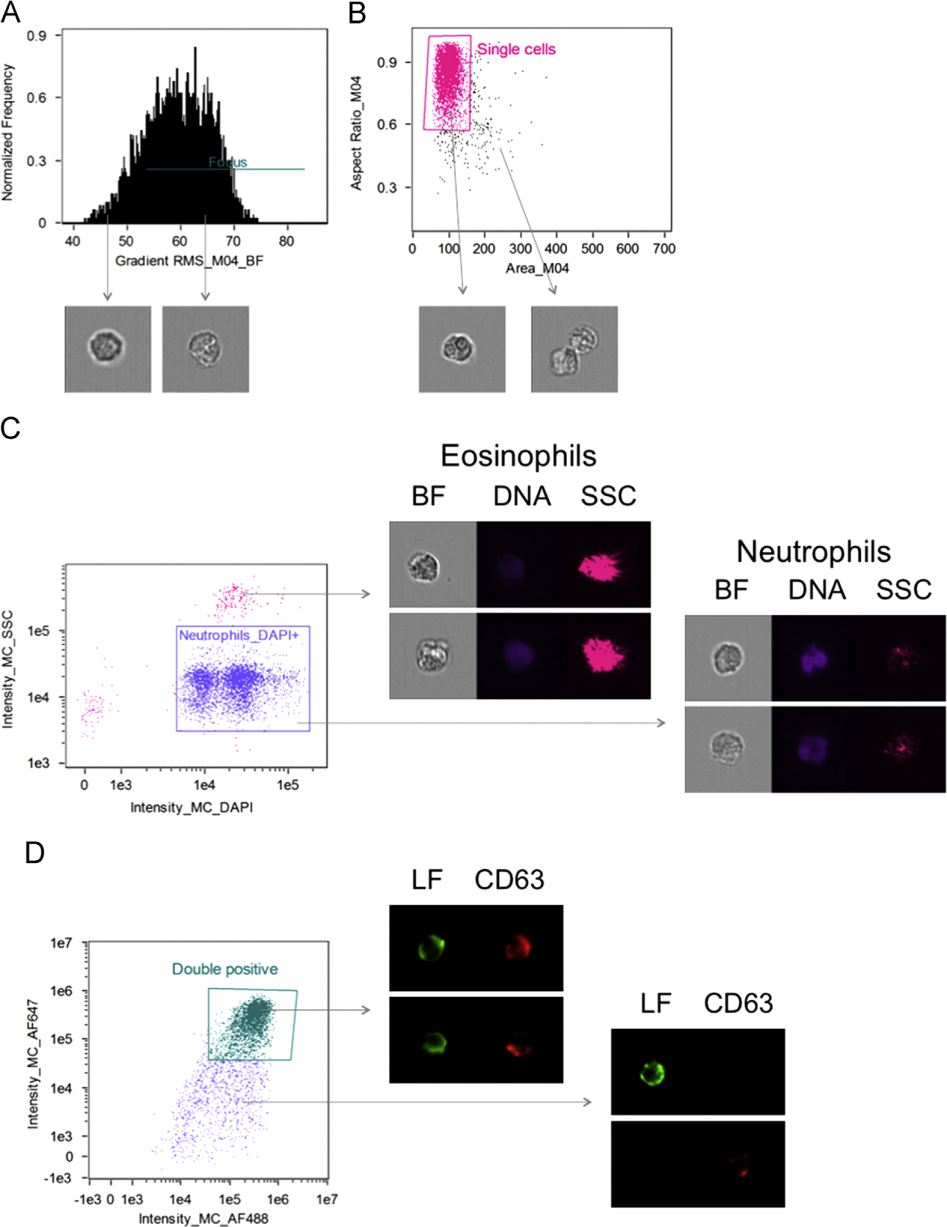
Gating strategy. The gating was done according to the colocalization wizard in the IDEAS software. Only cells in focus (A) and single cells (B) were included. Neutrophils were gated based on their positivity of DAPI and being low in side scatter (SSC), excluding anuclear debris and eosinophils (high in side scatter due to inherent aoutofluorescence) (C). Finally, only neutrophils with strong positive staining for both markers were selected (D). Brightfield (BF) images and indicated fluorescently labeled markers are depicted.

Since the objective of the experimental set-up was to perform quantitative image analyses of neutrophil granule markers, the following gating strategy (essentially as described in [22]) was employed to identify the correct cells. First, cells in focus were gated (Fig. 1A) followed by inclusion of single cells only (Fig. 1B). Next the neutrophils were gated to include only cells that were high in DAPI staining (to exclude anuclear debris) but low in side scatter (SSC) to exclude eosinophils (which are present in the granulocyte preparation and are high in SSC due to their inherent autofluorescence) from further analyses (Fig. 1C). Finally the colocalization analysis requires positive staining of both markers to be analyzed and thus only double positive events with rather intense staining were selected for subsequent analysis (Fig. 1D).

### 2.3 Colocalization

Included in the IDEAS software is a colocalization wizard that was used as guidance for the described analysis. The analysis has previously been used to study colocalization of for example different markers in phagosomes [23], [24] or autophagosomes [25], [26]. The wizard measures the degree to which punctate staining of two probes spatially colocalize in the collected images, and calculates a ‘bright detail similarity R3’ feature for each cell, defined as the log transformed Pearson׳s correlation coefficient of the localized bright spots with a radius of 3 pixels or less in two images [26]. The ‘median bright detail similarity R3’ feature of each sample was then calculated and this feature will be referred to as the colocalization score in the text below.

**Fig. 2:**
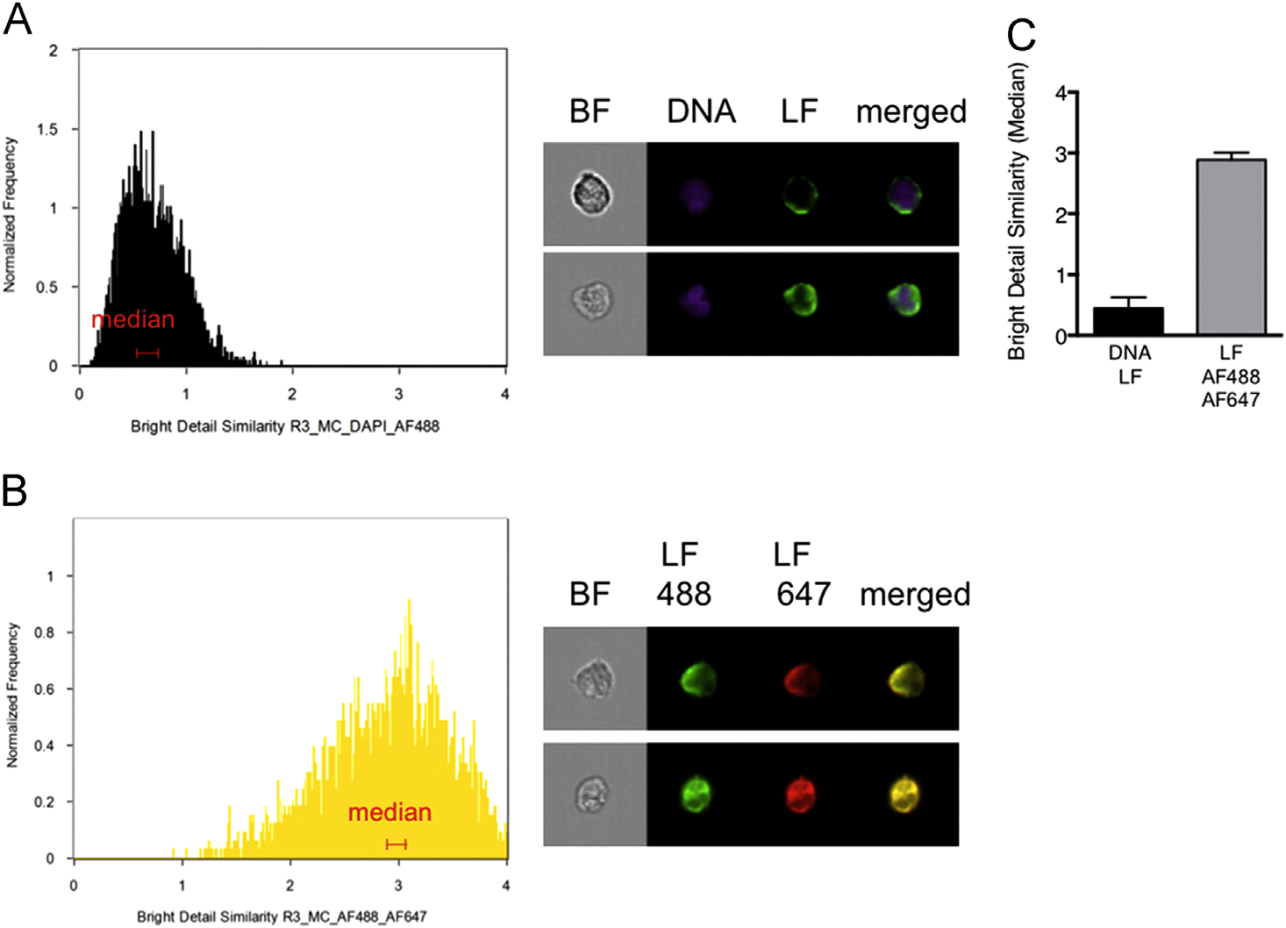
Negative and positive technical controls of colocalization. (A) The bright detail similarity R3 feature (colocalization score) for DAPI (DNA) and lactoferrin (LF) was used as a negative technical control with minimal colocalization. (B) Colocalization of two different secondary antibodies (conjugated with Alexa Fluor 488 or 647, respectively) against the lactoferrin antibody, was used to obtain the maximal possible colocalization (positive technical control). Histograms of representative samples and examples of cells found around the median colocalization score (ranges indicated in red on the histograms) are shown as brightfield (BF) images and indicated fluorescently labeled markers. (C) The mean colocalization score (determined from the median values from 3 independent experiments) +SD is shown.

The ‘bright detail similarity R3’ feature is expressed in arbitrary units and in order to get a better idea of what the colocalization score really represents, we first used a set of two technical controls of stained, resting neutrophils. The negative control was calculated as the colocalization of DNA (DAPI) and lactoferrin, where no colocalization is to be expected since lactoferrin is found in specific granules and not in the nucleus. The colocalization score for this negative control was 0.64 and as expected, representative images confirmed that the two markers were clearly separated spatially with minimal overlap (Fig. 2A). The positive (technical) control was a sample stained with one primary antibody against lactoferrin followed by two different secondary antibodies (with distinct fluorochromes). Theoretically it should not be possible to obtain a higher colocalization score than with two probes identifying the same structure (Fig. 2B), and the colocalization score for the positive technical control was 3.0 with representative images displaying complete spatial overlap. From these two technical controls the range of colocalization to be expected when analyzing two distinct granule markers was defined. Using mean colocalization scores from three independent experiments featuring the negative and positive technical controls, respectively (Fig. 2C), the range of colocalization was defined as around 0.5 (minimum colocalization) to 3 (maximum theoretical colocalization) arbitrary units.

### 2.4 Colocalization of neutrophil granule markers

**Fig. 3:**
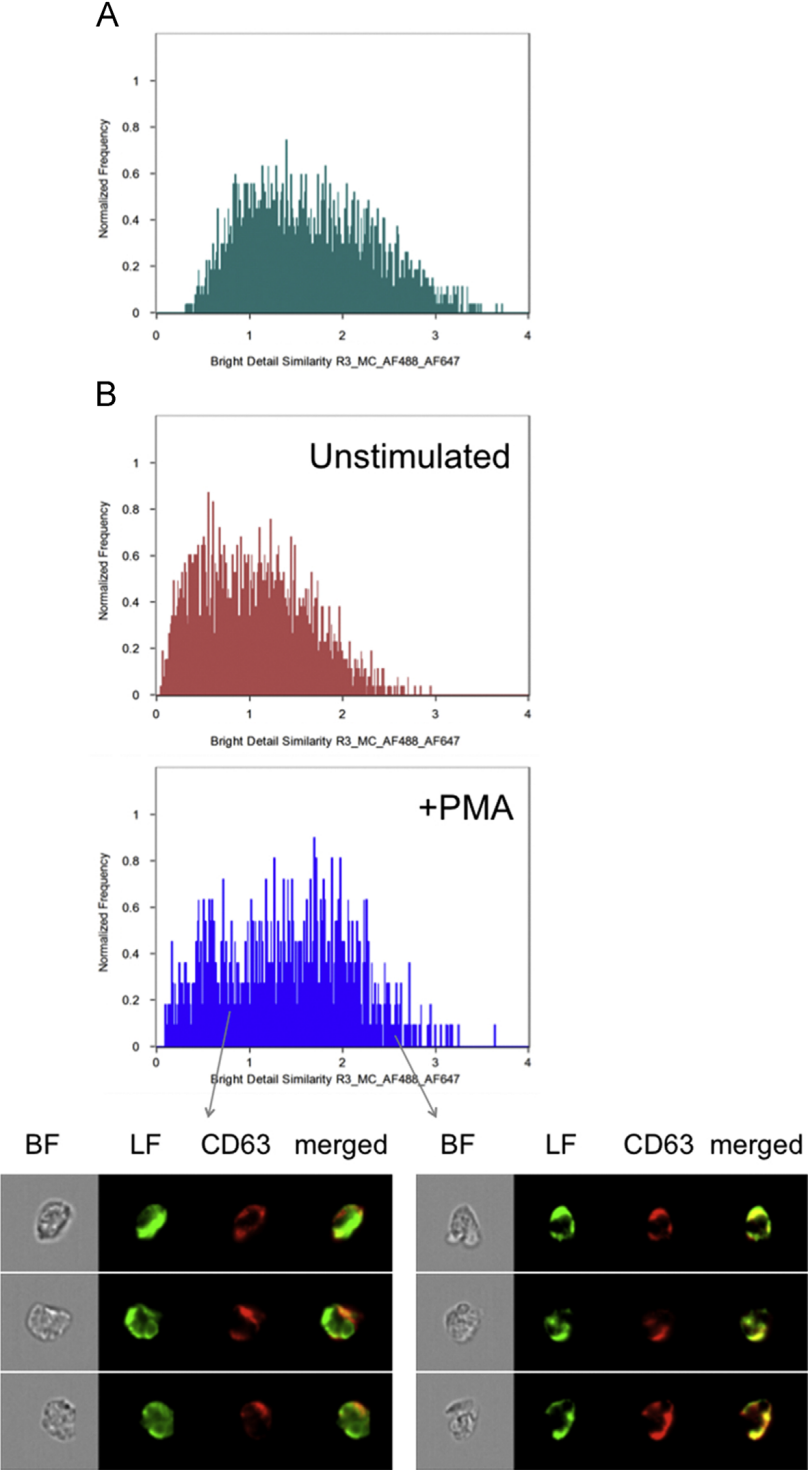
Colocalization of neutrophil granule markers. (A) Histogram depicting the colocalization of lactoferrin and NGAL, two markers of specific granules (positive biological control). (B) Histograms depicting the colocalization of lactoferrin (specific granules) and CD63 (azurophil granules) without (red) and with (blue) PMA stimulation. Examples of cells with low and high colocalization values (chosen from positions in the histogram as indicated by the arrows) in a sample of PMA-stimulated neutrophils stained for lactoferrin (green) and CD63 (red) are also shown.

The positive technical control used to determine maximum colocalization (Fig. 2B) is valuable for determining the theoretical maximal colocalization score. However, for a more biologically relevant control we also measured the colocalization of two markers that are known to reside in the same granule type. Both lactoferrin and NGAL are localized in the specific granules [5] and the colocalization score for these two markers in resting cells (Fig. 3A) was 1.6 which is within the range calculated above (Fig. 2C).

Heterotypic granule fusion between specific granules and azurophil granules has previously been observed microscopically after e.g., PMA stimulation [16], [27], [28]. In addition, such fusion has been indirectly concluded to take place based on the fact that PMA-stimulation gives rise to intracellular MPO-processing of ROS originating from the NADPH-oxidase [1], [17], [18], [19]. In order to objectively quantify heterotypic granule fusion we utilized the methodology described above to calculate colocalization scores between lactoferrin (marker for the specific granules) and CD63 (marker for the azurophil granules). In unstimulated cells the colocalization score for lactoferrin and CD63 was low, as expected, but after stimulation with PMA a clear shift was observed in the histogram was observed (Fig. 3B and [1]). In the attached data set, the colocalization score increased from 1.0 to 1.4 upon PMA stimulation (Supplemental data). Worth noting is that the PMA-stimulated sample contained cells with rather low colocalization values as well as cells with high values close to that of the positive control sample (for examples, see Fig. 3B). Whereas traditional image analysis of microscopic slides could easily be biased based on which cells are selected for analysis, the analysis described above provides an unbiased calculation of the degree of colocalization.

## Appendix A Supplementary material

Supplementary material

Supplementary material *Colocalization.cif* Raw data from one representative experiment, including the different control samples. This is a color compensated image data file that can be opened using IDEAS software when applying the corresponding .daf analysis file

Supplementary material *Colocalization.daf* A data analysis file containing a template for all the analysis steps described. When opened in IDEAS software, this file will display the data contained in the corresponding .cif file, analyzed for colocalization

## Footnotes

Appendix A Supplementary data associated with this article can be found in the online version at doi:10.1016/j.dib.2015.12.003.
